# Off-Label Use of Intravenous Immunoglobulin with Methylprednisolone to Treat Parsonage–Turner Syndrome in a United States Marine

**DOI:** 10.1155/2021/6663755

**Published:** 2021-04-01

**Authors:** Carissa M. Sedlacek, Michael Leone, Adam D. Foster, Amy Hinkelman

**Affiliations:** ^1^Campbell University, Jerry M. Wallace School of Osteopathic Medicine, P.O. Box, Buies Creek, NC 27506, USA; ^2^Neurology Department, Gundersen Health System, 1900 South Avenue, La Crosse, WI 54601, USA; ^3^Department of Anatomy, Campbell University, Jerry M. Wallace School of Osteopathic Medicine, P.O. Box 4280, Buies Creek, NC 27506, USA; ^4^Department of Microbiology and Immunology, Campbell University, Jerry M. Wallace School of Osteopathic Medicine, P.O. Box 4280, Buies Creek, NC 27506, USA

## Abstract

Neuralgic amyotrophy (NA) also known as Parsonage–Turner syndrome is an inflammatory disorder of the brachial plexus characterized by sudden, acute onset of severe pain of the arm and/or shoulder followed by muscle weakness and sensory abnormalities. Although management may involve physical therapy, immunomodulatory drugs, and analgesics, there is nothing specific for the treatment of NA. Full functional recovery can take months to years, but recurrence and/or persistence of symptoms and disability are frequent. This case reports a 22-year-old male who recovered from NA within 3 months following treatment with 1000 mg of methylprednisolone and off-label use of 0.5 g/kg of intravenous immunoglobulins (IVIG) for four consecutive days. Three years later, the patient experienced soreness and paresthesia of the shoulder following a military shooting exercise, and 0.75 g/kg of IVIG and 1000 mg of MP were prescribed for 2 consecutive days resulting in complete recovery and no recurrences to date. EMG findings, 3.5-year postinitial treatment, revealed improvement in the brachial plexopathy. This provides support for the combined use of IVIG and glucocorticoids in the treatment of NA and highlights the need for further studies investigating whether this combined treatment regimen may accelerate recovery and improve long-term outcomes for patients diagnosed with NA.

## 1. Introduction

Parsonage–Turner syndrome, also more commonly referred to as neuralgic amyotrophy (NA), inflammatory brachial plexopathy, or idiopathic brachial plexopathy, classically presents as sudden, severe, unilateral, neuropathic pain in the arm or shoulder followed by paresis, muscular atrophy, and sensory loss [1–4]. The initiation and progression of the disease is suspected to involve a combination of genetic, biomechanical, immunological, and environmental factors but can also be hereditary with an autosomal dominant inheritance or idiopathic [1, 3, 4]. Although historically considered a rare disease with estimates of 2-3 cases per 100,000/year in the general population [5, 6], the actual incidence rate is predicted to be much higher at 1 in 1000 patients among the general population when primary care providers received more training in diagnosing this disorder [6]. Similarly, the Dutch military suspects their current NA incidence rates, 18 cases per 100,000/year to be an underestimation due to lack of training and recognition of this disease by military personnel and physicians [7]. Despite increasing recognition and diagnosis of NA, recovery times are extensive and prognosis remains poor, highlighting the need to report alternative treatments with improved outcomes [1, 2, 6, 7].

Since there is no specific treatment for NA, traditional management involves an expectant, or “wait and see” approach, while more recent studies suggest a high-dose oral corticosteroid early in the disease process and a long-acting opioid and nonsteroidal anti-inflammatory drugs (NSAIDs) if pain is present [1, 3–5, 8, 9]. However, with these therapies, many patients remain in pain or have sustained loss of function; less than 10% of patients, in a 246-person cohort, reported a full recovery from NA after 3 years [10]. Recovery time is also extensive, lasting between 6 and 18 months with recurrence rates at 25% and 75% for idiopathic and hereditary NA, respectively [1, 10]. This highlights the need for more effective therapies to attenuate NA symptoms and promote functional recovery. In the following case report, a suspected immune-mediated pathogenesis of NA prompted treatment with intravenous immunoglobulins (IVIG), (Gamunex-C^®^, Research Triangle Park, NC) for its potential anti-inflammatory and immunomodulatory effects [11], and methylprednisolone acetate (MP) (Depo-Medrol^®^, Kalamazoo, MI). Administering this therapy during the NA acute phase elicited a full functional recovery within 3 months for this patient.

## 2. Case Presentation

An otherwise healthy, 22-year-old male Marine presented to the Neurology Department with painless, progressive paresthesia and paresis to his right dominant limb that started 14 days prior. During that time, he was participating in an 8-day training exercise where he hiked nearly 113 km carrying a total load of 54–60 kg. His family history was unremarkable, but his medical history was notable for right acromial numbness and paresthesia without paresis lasting three months with no medical intervention, following a 19 km run with a 20 kg rucksack, eight months prior.

Sixteen hours into his 8-day training exercise, he began experiencing painless, right arm weakness with no improvement when the pack was removed. By day 5, his shoulder and upper arm were paretic. He retained his forearm range of motion, but with notable weakness. On day 7, he was unable to perform fine motor tasks, and by day 9, he had approximately 4 kg of right-hand grip strength.

Initial physical examination revealed no aphasia or dysarthria, facial droop, or apparent higher cortical function deficits. His extraocular muscles were intact, as well as the range of motion of his cervical spine. All physical exam findings of the extremities were compared bilaterally with pertinent exam findings only noted on the patient's right upper extremity. All other extremities had normal findings.

He had mild winging of the right scapula, some atrophy of the right shoulder girdle, and deltoid but could shrug his shoulder. Right rhomboids and serratus anterior were intact. A few deltoid and triceps fasciculations were observed. Using Medical Research Council (MRC) criteria, motor scores on the patient's right side were as follows: deltoid 4, supraspinatus 3, pectoralis 4, biceps 4, brachioradialis 4, triceps 3, wrist extensors 2, finger extensors 2, pronator teres 4, finger flexors 4, intrinsic hand movement 4, flexor carpi ulnaris 3-4, and all contralateral muscles of the left upper extremity were 5 (Table 1). His right upper extremity was areflexic, and his left was 2+. Bilaterally, his patellar reflexes were 2+, ankle jerks were 2+, and toes were downgoing. The patient had lack of sensation to light touch and pinprick from the dorsum of his right shoulder and triceps, distally. Position sense was intact. He had no Romberg's sign, and his gait and station were normal.

**Figure 1:**
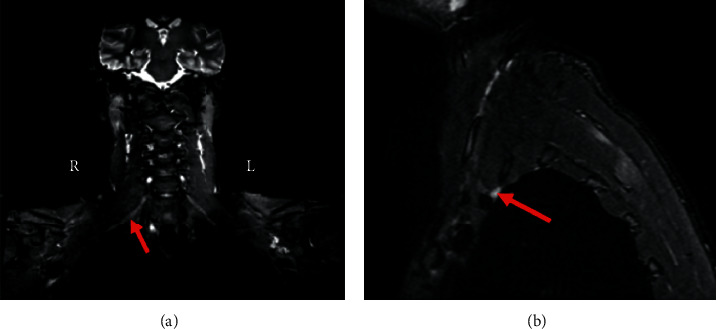
Chest MRI of the patient with inflammatory brachial plexopathy. (a) Coronal chest and (b) sagittal chest, T2 weighted and fat-suppressed MRI images without contrast show elevated T2 signaling indicating subtle thickening in the right brachial plexus structure at the time of diagnosis.

Chest MRI without contrast showed asymmetry of the nerve roots on the right side. Additionally, there was a subtle increase in T2 signal intensity and enlargement in the roots of C7, C8, and T1 in coronal and sagittal exams (Figures 1(a) and 1(b)). This enlargement extended into the middle, and inferior trunks. Compared to the contralateral side, the right brachial plexus divisions were enlarged with an increased T2 signal intensity.

An EMG was performed to further determine the extent of injury. Right brachial plexopathy involving primarily the posterior and lateral cords was confirmed by EMG. The deltoid, triceps brachii, biceps brachii, brachioradialis, and extensor digitorum communis in the right arm displayed acute, and to a lesser extent chronic, denervation changes. No electrophysiological evidence for nerve entrapment or radiculopathy was noted in the right upper extremity. These findings provided the basis for diagnosis of inflammatory brachial plexopathy.

The patient was treated for 4 consecutive days with 1000 mg of MP and 0.5 g/kg of IVIG. With this therapy, he experienced transient headaches, abdominal and facial edema, and diaphoresis. After the infusions were completed, he was given 80 mg of oral prednisone and instructions to taper the dosage by 20 mg every 3 days. The patient also received physical therapy for 4 months. Within 3 months, motor function and cutaneous sensations were fully recovered, and he was able to continue his military training.

Two years later, following a shooting exercise, the patient developed shoulder soreness and paresthesia in his right upper extremity. He was prescribed boosters of IVIG 0.75 g/kg and 1000 mg of MP for 2 consecutive days and the same course of oral prednisone. The paresthesia subsided upon completion of the treatment and has not recurred.

Upon posttreatment examination 3.5 years after the initial injury, consistent with initial findings, he had mild winging of the right scapula, and shoulder shrug, rhomboids, and serratus anterior remained intact. He had no Romberg's sign, and his gait and station were normal. Compared to the initial examination, any atrophy, fasciculations, or deficits in sensation were no longer apparent. Motor scores of the right side were generally improved: deltoid 5, supraspinatus 5, pectoralis 4, biceps 5, brachioradialis 5, triceps 4, wrist extensors 4, finger extensors 4, pronator teres 5, finger flexors 5, intrinsic hand movement 5, and flexor carpi ulnaris 4, and the left upper extremity 5 (Table 1). His right upper extremity reflex was 1+ (compared to previously being areflexic), and his left upper extremity reflex maintained at 2+. Bilaterally, his patellar reflexes 2+, ankle jerks 2+, and toes were downgoing, indicating no changes from the initial exam.

A follow-up EMG performed 3.5 years postinitial injury showed chronic, but not acute, denervation in muscles innervated by the superior trunk of the right brachial plexus, indicating an improvement in the brachial plexopathy (Tables 2 and 3).

## 3. Discussion

Neuralgic amyotrophy is a debilitating disease that can be initiated by genetics, biomechanical factors, immunological factors, or a combination of these factors [1, 12]. Septin-9 on chromosome 17q25, a protein responsible for cytoskeleton formation, cellular division, and tumorigenesis, is commonly mutated among those with hereditary NA, an autosomal dominant disorder [12]. Hereditary NA and idiopathic NA are clinically similar, but hereditary NA tends to have more recurrent attacks, extrabrachial attacks, and increased duration of pain and disability due to repeated attacks [12, 13].

Biomechanical and immunological events that may trigger NA include viral and bacterial infections, surgery, vaccinations, childbirth, strenuous exercise, injury, medical procedures, or autoimmune or connective tissue medical conditions [9,13]. This case report describes a US Marine that developed rucksack palsy, which served as a traumatic trigger in his development of NA. Despite his poor prognosis, he achieved a complete functional recovery following treatment with IVIG and MP [1, 10]. Evidence of acute inflammation and axonal loss on his MRI and EMG made him a prospective candidate for this therapy, as there is evidence to support NA as an organ-specific immune-mediated disorder, rather than a generalized autoimmune disorder [1, 14].

Immune changes seen in patients with NA include blastogenic activity of lymphocytes within the brachial plexus [1, 14, 15], presence of antiganglioside antibodies [10, 16], increased serum antiperipheral nerve myelin antibodies, and soluble terminal complement activation products during the acute phase of NA [17]. Based on these data of prior NA patients, there are several, theorized mechanisms of action of how IVIG may have contributed to the healing process and full functional recovery in this patient. IVIG has regulatory effects on the complement system such as inhibiting active complement components from binding to and initiating destructive inflammatory responses against tissues; IVIG can also neutralize the anaphylatoxins, C3a and C5a, thereby reducing inflammation in NA [18, 19]. IVIG may also accelerate the clearing of pathogenic IgG seen in NA [20].

Other NA case studies treated with IVIG and MP have demonstrated improved outcomes, often with shortened recovery times, compared to expectantor MP management [5, 16, 21–25]. However, it has not been shown to improve the prognosis for every patient. This necessitates further research to better understand the mechanisms of NA and diagnostic indicators warranting this treatment regimen.

## 4. Conclusion

This case report demonstrates the complete functional recovery of a patient suffering from NA following treatment with IVIG and MP within 3 months. These results are noteworthy as full functional recovery is rarely obtained with current recommended therapies, and partial functional recovery may take 6–18 months. The detection of antiganglioside antibodies and other inflammatory mediators prompted clinicians to consider IVIG and MP for the treatment of NA in this case. The efficacy and best time to administer this therapy has yet to be determined, but there are several cases that support the idea that IVIG and MP alleviate the symptoms and often improve the prognosis of NA. Although further research is needed to better understand the pathogenesis of NA and the therapeutic mechanism, efficacy, and potential adverse effects of IVIG in the treatment of NA, these results support a promising additional indication and usage for IVIG.

## Figures and Tables

**Table 1:** Summary of Medical Research Council (MRC) scores of right limb before and after IVIG treatment.

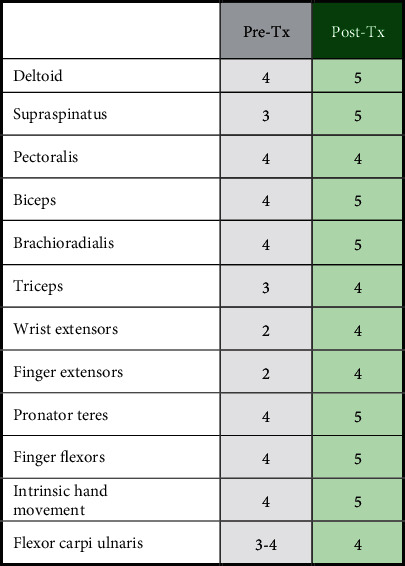

Summary of MRC scores comparing the right limb at the time of diagnosis (Pre-Tx, gray shading) to MRC scores obtained 3.5 years posttreatment to evaluate chronic changes (Post-Tx, green shading).

**Table 2:** Summary of EMG findings of right limb before and after IVIG treatment.

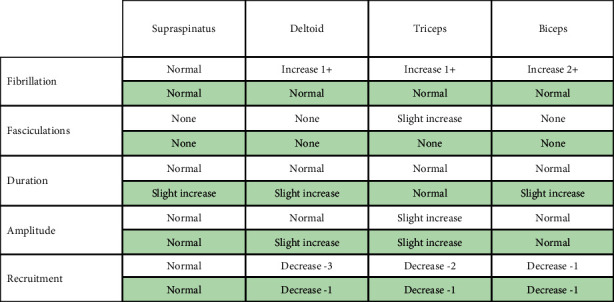

Summary of EMG findings in the right limb at the time of diagnosis (gray shading) compared to the posttreatment EMG (green shading), 3.5 years later. At both timepoints, normal EMG findings were noted for the right flexor carpi ulnaris and pronator teres (not shown).

**Table 3:** Nerve conduction findings of right limb.

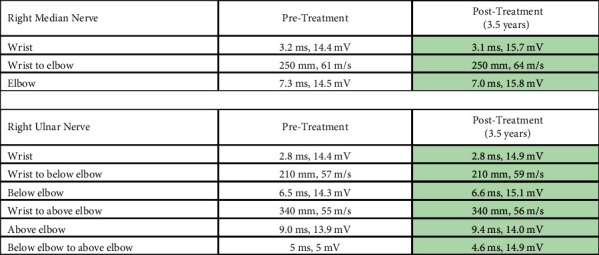

Summary of nerve conduction findings of the right limb at the time of diagnosis (pretreatment, gray shading) compared to 3.5 years, posttreatment with IVIG (posttreatment, green shading).

## Data Availability

The data used to support the findings of this study are available from the corresponding author upon request.

## Additional Points

Following my diagnosis of Parsonage–Turner syndrome, it was hard for me to believe that I had a chance at fully recovering based on my initial presentation. Over the course of 8 days carrying a heavy rucksack, my right arm went from a muscular, fully functioning arm to a partially sensationless and atrophied shoulder that would not move unless I picked it up with my left arm. Based on this presentation, I knew my military career would be over unless I could regain the function of my arm. IVIG and MP were offered to me with an understanding that this was an off-label use of the IVIG. I felt the potential benefits of the therapy outweighed the risks. I completed my course of IVIG and MP and remained compliant with the physical therapy exercises. Within 3 months, I regained full function and sensation in my right arm. Within 4 months, I rebuilt the strength in my right arm to be able to lift and shoot my rifle in training for longs periods of time. Most importantly, I regained the ability to use my arm again, which enabled me to continue my military career and support my family.
